# Anti-Obesity and Weight Management-Related Antioxidant Potential Properties of Calabrian Pine Extracts: *Pinus nigra* Subsp. *laricio* (Poir.) Maire

**DOI:** 10.3390/plants14060851

**Published:** 2025-03-08

**Authors:** Mary Fucile, Carmine Lupia, Martina Armentano, Mariangela Marrelli, Ludovica Zicarelli, Claudia-Crina Toma, Giancarlo Statti, Filomena Conforti

**Affiliations:** 1 Department of Pharmacy, Health and Nutritional Sciences, University of Calabria, 87036 Rende, Italy; mary.fucile@unical.it (M.F.); martina.armentano@libero.it (M.A.); mariangela.marrelli@unical.it (M.M.); ludovica.zicarelli@unical.it (L.Z.); giancarlo.statti@unical.it (G.S.); 2 Mediterranean Ethnobotanical Conservatory, 88054 Sersale, Italy; studiolupiacarmine@libero.it; 3 National Ethnobotanical Conservatory, 85040 Castelluccio Superiore, Italy; 4 Department of Pharmacognosy, Faculty of Pharmacy, Western University “Vasile Goldis” of Arad, L. Rebreanu Street, No. 87, 310048 Arad, Romania; claudiatoma2004@yahoo.com

**Keywords:** *Pinus*, lipase, amylase, apical shoots, branches

## Abstract

Natural extracts derived from plants have gained attention as potential therapeutic agents for obesity management. Some natural extracts were demonstrated to inhibit pancreatic lipase and alpha amylase, potentially influencing nutrient absorption and contributing to weight management. *Pinus nigra* subsp. *laricio* (Poir.) Maire, commonly known as the Calabrian pine or larch pine, is a subspecies of the black pine native to the mountains of southern Italy and Corsica. This study investigated the phytochemical content and antioxidant (DPPH and β-carotene bleaching assays) and enzymatic (lipase and amylase inhibition) activities of ethanolic extracts from apical shoots and branches, fractionated into *n*-hexane, dichloromethane, and ethyl acetate. All the extracts were also subjected to a preliminary evaluation of their anti-inflammatory potential by measuring the ability to inhibit nitric oxide (NO) production in RAW 264.7 macrophages. The ethyl acetate branch fraction exhibited the strongest antioxidant activity (DPPH IC50 15.67 ± 0.16 μg/mL), while the total branch extract best inhibited pancreatic lipase (IC50 0.62 mg/mL). Amylase inhibition was strongest in the ethyl acetate apical shoot fraction (IC50 22.05 ± 0.29 µg/mL). The branches’ hexane and dichloromethane fractions showed the greatest anti-inflammatory potential, inhibiting NO production in RAW 264.7 cells with IC50 values comparable to the positive control.

## 1. Introduction

*Pinus nigra* J.F. Arnold (European black pine) is native to Europe and Asia. Two different subspecies exist in Italy: *Pinus nigra* subsp. *laricio* (Poir.) Maire, commonly known as larch pine, and *P. nigra* subsp. *nigra* (black pine) [1]. The *P. nigra* subsp. *laricio* (Poir.) Maire is endemic to Italy and occurs naturally in Sicily and Calabria, with the greatest distribution in the Silan Plateau. In Sicily, it grows on the volcanic soils of Etna: numerous specimens are found in the Linguarossa pine forest [2]. The larch pine occupies a significant position in the historical narratives of ancient and modern Mediterranean civilizations. Indeed, it is considered an iconic botanical presence in Calabrian Mountain forests. This plant was well known, for example, to the ancient Romans, who valued its timber for construction, especially for making beams and ships. Additionally, the larch pine was renowned for its exceptional resin, known as pitch brutia. This aromatic resin was highly sought after in the ancient world, celebrated as the most valuable and fragrant of its kind [3].

Obesity is a chronic global health concern characterized by excessive body fat accumulation, leading to a plethora of adverse health consequences, including diabetes, hypertension, dyslipidemia, cancer and increased oxidative stress. This condition is marked by chronic low-grade inflammation, a major source of ROS (Reactive Oxygen Species) production. One mechanism underlying these damaging effects is the dysregulation of oxidative balance, which results in increased oxidative stress. Adipose tissue, the primary site of fat storage, releases inflammatory mediators that activate immune cells, further increasing ROS generation [4].

Lipase and amylase inhibitors represent a potential class of anti-obesity agents that target key digestive enzymes involved in nutrient breakdown and absorption. Lipase inhibitors act by binding to the active site of lipase, thus blocking its ability to hydrolyze triglycerides. This results in the excretion of undigested fats in the feces, leading to reduced caloric absorption. Amylase inhibitors block the breakdown of carbohydrates, preventing the absorption of simple sugars into the bloodstream. This can lead to reduced blood glucose levels and decreased overall caloric intake. A growing body of evidence suggests that natural compounds derived from plants may offer therapeutic benefits for various health conditions. Recent studies have highlighted the potential of pine needle extracts as a source of bioactive compounds with anti-obesity properties. For instance, a study by Kim et al [5], showed that pine needle extract can stimulate the activity of proopiomelanocortin neurons in the hypothalamus, a crucial brain region in the regulation of appetite and energy metabolism. In addition, treatment with the extract led to a reduction in body weight, fat mass, and plasma leptin levels, as well as improved glucose metabolism in obese animal models. Notably, pine needle extract selectively inhibited lipogenesis in white adipose tissue without significantly affecting thermogenesis in brown adipose tissue [5]. The therapeutic benefits and traditional uses of pine extracts have been recorded worldwide. In ancient times, essential oils derived from pine needles found wide use in folk medicine to treat cardiovascular conditions and fight respiratory infections [6]. Recent studies have demonstrated the antioxidant and antimicrobial activity of essential oils from needles [7,8] and from hydroalcoholic extracts from needles and bark [9]. In traditional medicine, pine bark has been widely used for wound care, management of inflammatory diseases, skin problems, and for the treatment of scurvy. Currently, pine bark extracts are identified as a fascinating and potent source of natural polyphenols with various biological activities. Pine extracts sold as phytochemical remedies are available on the market. One such example is Pycnogenol™, a standardized bark extract derived from *P. maritima*. This extract boasts a wide range of biological activities and is used in the treatment of conditions related to chronic inflammation and circulatory dysfunction [10]. Pine extracts are rich in bioactive compounds, including a wide range of phenolic compounds. These compounds, characterized by the presence of an aromatic ring linked to at least one hydroxyl group (-OH), impart antioxidant, anti-inflammatory, and antimicrobial properties to the plants. Detailed phytochemical analyses have revealed the presence of stilbenes, such as pinosylvin and its methylated derivatives, in pine wood extracts. The chemical structure of these compounds, characterized by an extended conjugate system, gives them distinct antioxidant properties due to their ability to donate electrons and stabilize free radicals. In a study by Perri et al [11], an extract obtained from larch pine wood significantly inhibited the release of pro-inflammatory cytokines (TNF-α and IL-6) and the NO mediator in LPS-stimulated RAW 264.7 cells. In addition, the extract showed downregulation of phosphorylated JAK2 and STAT3 proteins [11].

The purpose of this investigation was to determine the antioxidant activity of the shoots and apical branches of *Pinus nigra* subsp. *laricio* (Poir.) Maire, as well as potential anti-obesity activity through the inhibition of pancreatic lipase and α-amylase. All the extracts were subjected to a preliminary evaluation of their anti-inflammatory potential by measuring their ability to inhibit nitric oxide (NO) production in the macrophage cell line RAW 264.7. For the phytochemical profile, an evaluation of the apolar compounds by gas chromatography was performed, and the total phenolic content and flavonoid content were also determined.

## 2. Results

### 2.1. Phytochemical Profile

The apical shoots and branches were extracted with ethanol by maceration. The percentage extraction yields, expressed on g of dry plant material, were calculated. The yields obtained were 10.88% and 6.40% for the two crude extracts, respectively (Table 1). A fraction of each crude extract was then successively extracted with solvents of different polarity: *n*-hexane (5.14% and 1.22% for apical shoots and branches, respectively, yield referring to dry plant material), dichloromethane (2.20% and 0.35%), and ethyl acetate (0.69% and 0.36%). The remaining aqueous fractions (2.85% and 4.47%) were also analyzed.

The total phenolic and flavonoid content were also assessed; the amounts were expressed as chlorogenic acid and quercetin equivalents per g of dry material, respectively. The highest content was found for apical shoots, both for total phenolic compounds and total flavonoids (Table 1).

The phytochemical content of the apolar fractions was assessed by means of GC-MS. The *n*-hexane samples were particularly rich in fatty acids, especially unsaturated fatty acids (Table 2). Elaidic acid and vaccenic acid (6.2% and 3.7% of the total peak areas in the total ion current (TIC)) were the most abundant for the branches.

In the dichloromethane fraction, the major component of the apical casts was found to be stigmast-3-en-6-ol, the only identified phytosteroid, with a percentage of 3.2. Fatty acid esters such as the methyl and ethyl esters of palmitic acid were also identified. For the branch extract, the percentages of the compounds were all less than 1 percent. The presence of a flavonoid with strong antioxidant properties, pinocembrin was detected albeit with a low percentage of 0.5% (Table 3). The only other compounds found were vanillic alcohol and isomaturnin.

### 2.2. Antioxidant Activity

The antioxidant potential of the extracts was assessed using the DPPH radical scavenging assay and the β-carotene bleaching assay. The results of the free radical scavenging activity of the different extracts are shown in Table 4. All the branch extracts showed dose-dependent activity. For the apical shoots, only the raw extract and the ethyl acetate fraction showed activity at the concentration tested. The best activity was found for the ethyl acetate fraction of both samples, followed by the raw extract. At the highest concentration (1 mg/mL) both ethyl acetate fractions showed an excellent inhibition percentage (97.22 ± 1.17% for the apical shoots and 95.80 ± 1.25% for the branches).

The best anti-scavenging activity was observed for the branches, particularly for the ethyl acetate fraction with an IC_50_ value of 15.67 ± 0.16 μg/mL, followed by the raw extract (IC_50_ = 26.14 ± 0.90 μg/mL).

In vitro lipid peroxidation inhibition was assessed using the β-carotene bleaching assay. This method involves measuring the β-carotene discoloration caused by linoleic acid degradation products at elevated temperatures. Except for the *n*-hexane fraction and the aqueous fraction of apical shoots, all the extracts exhibited lipid peroxidation inhibition. After 30 min of incubation, remarkable activity was observed for the total branch extract, demonstrating an IC_50_ value of 3.33 ± 0.16 µg/mL, followed by the ethyl acetate fractions of both samples, which have, respectively, for the apical shoot and the branch extracts an IC_50_ values of 10.69 ± 0.26 µg/mL and 11.35 ± 015 µg/mL. After 60 min of incubation, the best activity was again demonstrated by the total branch extract with an IC_50_ value of 5.07 ± 0.15 µg/mL, followed by the ethyl acetate fraction of both extracts (Table 4).

### 2.3. Inhibition of Nitric Oxide Production

Inflammation is a complex response of the body to harmful stimuli, such as pathogens and oxidative stress. Macrophages, key cells of the immune system, play a central role in triggering and modulating this response. They phagocytize pathogens and damaged cells, releasing pro-inflammatory cytokines (such as TNF-α and IL-1β) that amplify the inflammatory response [12]. The extracts were also tested on nitric oxide (NO) production in the RAW 264.7 murine macrophage cell line. Macrophages were treated with varying concentrations of plant extracts (6.25–1000 μg/mL) in the presence of bacterial lipopolysaccharide (LPS) at 1 μg/mL for 24 h. The nitric oxide production was quantified using the chromogenic Griess reaction, which measures the accumulation of nitrite, a stable NO metabolite. The quantification of NO production was preceded by the MTT assay to assess the possible cytotoxic effects of the different concentrations of the extracts. Concentrations found to be cytotoxic to the cell line were not considered.

Once again, the best activity was observed for extracts from the branches. At the highest concentration tested that showed no cytotoxic effects (100 µg/mL), the hexane fraction inhibited nitroxide production with a % of 63.06 ± 1.54, while the dichloromethane fraction showed a % inhibition of 64.28 ± 5.28. As shown in Table 5, for apical shoots, only the hexane fraction showed activity. The best inhibition of NO production was obtained from the hexane and dichloromethane fractions of the branches, which reported an IC_50_ value comparable with that of the positive control used.

### 2.4. Pancreatic Lipase and α-Amylase Inhibition

**Figure 1:**
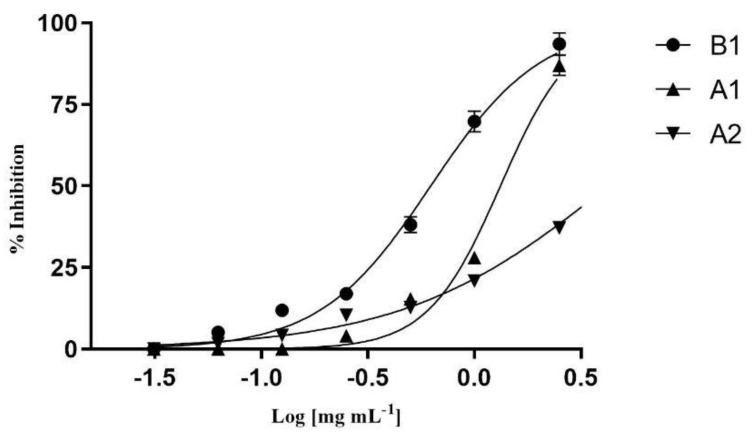
Concentration-dependent lipase inhibitory activity of B1: branch total extract; A1: apical shoot total extract; A2: apical shoot water fraction.

The anti-obesity potential of the extracts and their fractions were assessed by measuring the inhibition of two enzymes involved in the metabolism of lipids and carbohydrates. The inhibitory effect on porcine pancreatic lipase (type II) activity was assessed using p-nitrophenyl caprylate (NPC) as the substrate. Due to low fraction yields, only the total extracts and their aqueous fractions were analyzed. All the samples tested exhibited dose-dependent inhibition of the enzyme. A significant correlation was observed between the antioxidant properties of the extracts and their inhibitory effects on pancreatic lipase activity. Among all the samples, the total branch extracts, which showed the best lipid peroxidation, also demonstrated the best inhibition of pancreatic lipase, with an IC_50_ value of 0.62 mg/mL (Table 6), with a percentage of enzyme inhibition of 93.52 ± 3.45 at the highest concentration tested (2.5 mg/mL), while the apical shoots showed an inhibition of 86.97 ± 3.07 percentage at the same concentration (Figure 1).

The inhibition of α-amylase by the samples under examination was evaluated by measuring maltose production through the reduction of 3,5-dinitrosalicylic acid to 3-amino-5-nitrosalicylic acid; the colorimetric reaction corresponds to a color change from orange yellow to brick red, measurable at a wavelength of 540 nm. Acarbose was used as a positive control. As shown in Table 7, this time, only the apical shoots showed activity. All the fractions were shown to inhibit the enzyme, and the best IC50 value was obtained for the fraction in ethyl acetate (22.05 ± 0.29 µg/mL), followed by the water fraction (47.32 ± 1.43 µg/mL).

## 3. Discussion

Our results reveal promising biological activities for larch pine extracts, particularly regarding their antioxidant capacity and inhibition of key enzymes related to obesity. Analysis of the total phenolic and flavonoid content revealed the highest concentrations in the apical shoots. The phenolic content of the extracts aligns significantly with that in previous work, despite using a different methodology. As reported by Nisca and colleagues (2021) in a previous study [13], ultrasonic and microwave extraction produced hydroalcoholic extracts of *P. nigra* bark rich in phenolic compounds. The phenolic content was found to be comparable, despite the use of different plant parts and different extraction methodology. This suggests that extracts obtained from *P. nigra* could be a reliable source of phenolics for various applications. These results may still suggest that the total phenolic content of *P. nigra* bark may be influenced more by the plant matrix itself than by the specific extraction methodology. Therefore, further investigations will be needed to confirm this hypothesis and assess whether different methods influence the quantitative profile of the extracted phenolic compounds. An interesting profile of antioxidant activity was shown by the plant extracts analyzed, with some fractions standing out for their effectiveness in counteracting both free radicals and lipid peroxidation. In general, there was a tendency for the fractions obtained from the branches to show greater antioxidant activity than those derived from the apical shoots. Several studies have shown a structure–activity relationship for phenolic compounds, suggesting that the specific structure of these compounds may influence their antioxidant activity [14,15]. While a correlation between antioxidant activity and total phenolic content is often reported, our results indicate a nonlinear and inconsistent relationship between antioxidant activity and the amount of phenolic compounds and flavonoids. In a previous study, Apetrei and colleagues (2011) [9] evaluated the antioxidant activity of *Pinus cembra* hydroalcoholic extracts and found a high DPPH radical scavenging power of 86.7% for bark and 72.2% for needles at a concentration of 333.33 μg/mL. 

Beyond the antioxidant properties, different studies have highlighted the potential anti-obesity effects of pine extracts. One study [16] investigated the effects of an extract of the needles that demonstrated anti-obesity and cholesterol-lowering effects through activation of AMP-dependent protein kinase (AMPK). A second study showed that the aqueous extract of the needles could protect liver cells from oxidative stress in vitro and enhance the activity of antioxidant enzymes while reducing oxidative damage in the liver and kidneys of obese mice [17]. Oxidative stress plays a crucial role in the development of obesity-related complications, including insulin resistance and fatty liver disease. Therefore, the use of extracts with antioxidants as well as anti-lipidemic effects could help mitigate these complications. Extracts of different pine species have been shown in previous studies to be effective in alleviating comorbidities associated with obesity, such as hyperlipidemia and diabetes [18,19,20]. In this context, the study of the inhibition of enzymes involved in lipid and carbohydrate metabolism emerges as a valuable strategy to counter obesity. In the present study, the capacity of extracts derived from apical shoots and branches to inhibit pancreatic lipase and α-amylase, key enzymes in lipid and carbohydrate metabolism, was evaluated for the first time. Of note is the correlation observed between antioxidant activity and pancreatic lipase inhibition. The total branch extract, which demonstrated the highest antioxidant activity and inhibition of lipid peroxidation, also showed the most pronounced inhibition of pancreatic lipase. The observed concordance supports evidence in the literature of a link between the ability to mitigate oxidative stress and the modulation of lipid metabolism [21,22]. It is plausible that compounds responsible for the observed antioxidant activity, such as polyphenols, may also influence enzyme activity, thus paving the way for potential applications in weight management and in the prevention of associated metabolic disorders [23]. Another noteworthy observation is the differential activity demonstrated against α-amylase. While the inhibition of pancreatic lipase was most evident in branch extracts, the greatest α-amylase inhibitory activity was found for the apical shoots. This differential distribution of inhibitory activity may reflect the presence of distinct bioactive compounds, each of which possesses a different affinity for the active site of a specific enzyme. However, the potential inhibitory effect of the ethyl acetate fraction of apical shoots on pancreatic lipase deserves further investigation, as it could not be assessed in the present study. These results suggest that the investigated plant extracts, particularly the branch total extract for pancreatic lipase inhibition and the apical shoot fractions for α-amylase inhibition, possess inhibitory potential against enzymes involved in lipid and carbohydrate metabolism. The inhibition of these enzymes may have significant implications for weight management and for the prevention of obesity-related metabolic disorders. These preliminary data lay the foundation for deeper understanding. 

Future studies should focus on identifying the specific compounds responsible for the enzyme inhibitory activity observed against both pancreatic lipase and α-amylase and elucidating their molecular mechanisms of action. It would be interesting to investigate the precise nature of the inhibitory mechanisms: whether the inhibition is competitive for binding to the active site or noncompetitive and, thus, acts by inducing conformational changes that reduce or abolish the activity of the enzyme. In summary, our results highlight the significant application potential of larch pine extracts in the context of weight management and prevention of metabolic comorbidities associated with obesity. These data open the way for promising applications in the field of weight management and prevention of obesity-related metabolic disorders. Further investigation will deepen the understanding of the therapeutic potential of larch pine extracts and may help open up prospects of considerable interest for their application.

## 4. Materials and Methods

### 4.1. Chemicals

Folin–Ciocalteu reagent, aluminum chloride, sodium carbonate, 2,2-diphenyl-1-picrylhydrazyl (DPPH), ascorbic acid, Griess reagent (1% sulfonamide and 0.1% N-(1-naphtyl)ethylenediamine dihydrochloride in 2.5% H3PO4), 3-(4,5-dimethylthiazol-2-yl)-2,5-diphenyltetrazolium bromide (MTT), Dulbecco’s modified Eagle’s medium (DMEM), l-glutamine, fetal bovine serum (FBS), antibiotic/anti-micotic solution (penicillin/streptomycin), lipopolysaccharide (LPS), indomethacin, lipase type II from porcine pancreas, 4-nitrophenyl caprylate (p-NPC), TRIZMA base, orlistat, a-amylase from porcine pancreas, starch from potato, sodium potassium tartrate, sodium hydroxide, 3,5-dinitrosalicylic acid, phosphate-buffered saline (PBS), and acarbose were purchased from Sigma-Aldrich S.p.A. (Milan, Italy). All the solvents used were purchased from VWR International s.r.l. (Milan, Italy).

### 4.2. Plant Material and Sample Preparation

The samples were collected at Sila National Park (Calabria, Italy). The apical shoots were harvested in May, during the growing season, and the branches in July. A voucher specimen (leg. det. Carmine Lupia) was deposited at Mediterranean Etnobotanical Conservatory, Sersale, Catanzaro (position number 22 of the Pinaceae section). Both samples (190 g) were cut into small pieces, dried, and then extracted with ethanol through maceration (48 h × 3 times) at room temperature (plant/solvent ratio 1:8 g/mL). The total extracts obtained were filtered and dried under reduced pressure. A portion of the raw extract (6 g) was suspended in 100 mL of a 9:1 methanol/water solution and partitioned with an equal volume of *n*-hexane until exhaustion. The remaining extract was brought to dryness and then redissolved in 100 mL of water. The resulting aqueous solution was subjected to the same partitioning procedure with dichloromethane and then with ethyl acetate. The percentage extraction yields of the ethanolic extract and all the fractions were calculated with respect to the weight in grams of dry plant material. The apolar fractions (*n*-hexane and dichloromethane) were analyzed by GC-MS.

### 4.3. Total Phenolic and Flavonoid Content

The total phenolic content was determined by adding 1 mL of Folin–Ciocalteu reagent and 1 mL of Na_2_CO_3_ (7.5% *w*/*v*) to 200 µL of samples at a concentration of 2 mg/mL in a mixture of acetone/methanol/water/acetic acid (40:40:20:0.1) after incubation for 1 h at 60 °C. The absorbance was measured at 726 nm two hours later [24]. The flavonoid content was measured by adding 1 mL of 2% AlCl_3_ to 1 mL of sample having a concentration of 2 mg/mL in 80% EtOH. The absorbance was measured after 15 min at 430 nm. All the analyses were performed in triplicate. The values were interpolated with the calibration curves of standards, using chlorogenic acid and quercetin for polyphenols and flavonoids, respectively. The results are expressed as mg equivalent standards/g of dry plant material [25].

### 4.4. GC-MS Analysis

GC-MS analyses were performed for the *n*-hexane and dichloromethane fractions of each sample, using a gas chromatograph (Hewlett-Packard, Model 6890, Milan, Italy) coupled with a selective mass detector (Hewlett Packard Model 5973, Milan, Italy). A 10 µL GC microsyringe (Hamilton, Bonaduz, Switzerland) was used to inject samples (1 µL), and the instrument was equipped with an SE-30 capillary column of 100% dimethylpolysiloxane (30 m length, 0.25 mm in diameter, and 0.25 μm film thickness). The carrier gas used was helium. A programmed temperature scheme with an increasing temperature from 60 °C to 280 °C and a rate of 16°/min was used (total GC elution time of 20 min). The column inlet was set at 250 °C. The MS operating parameters were as follows: ion source, 70 eV; ion source temperature, 230 °C; electron current, 34.6 μA; vacuum 10^-5^ torr. The mass spectra were acquired over a 40–800 amu range at 1 scan/s. Helium was used as the carrier gas (linear velocity 0.00167 cm/s). Compounds were tentatively identified by comparing the obtained spectra with those of the Wiley Mass Spectral Database of the GC-MS system.

### 4.5. In Vitro Methods for Antioxidant Activity Assessment

Antioxidant activity was evaluated using two different methods: the DPPH test and the β-carotene bleaching test. To investigate the radical scavenging activity of the extracts, the 2,2-diphenyl-1-picrylhydrazyl (DPPH) test was performed [25]. The raw extracts and their fractions were serially diluted to final reaction volume concentrations between 10 and 1000 µg/mL, and 0.2 mL of each was added to 0.8 mL of a methanolic solution of DPPH 0.1 mM. Ascorbic acid was used as a positive control. After 30 min of incubation in the dark, the absorbance was measured at 517 nm. The DPPH radical removal activity is calculated by the following equation:

$$\%\;DPPH\;radical\;scavenging=\frac{sample\;absorbance}{control\;absorbance}\times 100$$

The β-carotene bleaching test is based on the decolorization of β-carotene, which is oxidized by the degradation products of linoleic acid by temperature. The addition of a substance that inhibits the degradation of β-carotene keeps the absorbance values stable [26]. A β-carotene solution (0.5 mg/mL) was prepared by dissolving 5 mg of β-carotene in 10 mL of chloroform. Next, 1 mL of this solution was added to 30 µL of linoleic acid and 300 µL of 100% Tween 20. The mixture was evaporated at 40 °C with a rotary evaporator to remove the chloroform, diluted with 100 mL of distilled water, and shaken vigorously to form an emulsion. Then, 5 mL of the emulsion was transferred into test tubes containing 0.2 mL of extracts and fractions at different concentrations (1–100 µg/mL). The tubes were then shaken gently and placed at 45 °C in a water bath for 60 min. The absorbance of the samples, positive control, and control group was measured at 470 nm using a Perkin-Elmer Lambda 40 UV/VIS spectrum. A blank, consisting of an emulsion without β-carotene, was used. Propyl gallate was used as positive control. Measurements were taken at the initial time (t = 0) and then at 30 and 60 min. All the samples were analyzed in triplicate, and the mean was calculated. The antioxidant activity (*AA*) was measured in terms of β-carotene bleaching using the following equation:

$$AA=\left(1-\frac{A_{0}-A_{t}}{{A{}^{\circ}}_{0}-{A{}^{\circ}}_{t}}\right)\times 100$$

where $A_{0}$ and ${A{}^{\circ}}_{0}$ are the absorbance values measured at the incubation time at time 0 for the samples/standard and control, respectively; ${A{}^{\circ}}_{0}$ and ${A{}^{\circ}}_{t}$ are the absorbance values of the samples/positive control and control group, respectively, at *t* = 30 and 60 min. The IC_50_ values for both tests were determined by plotting sample concentrations against the corresponding percentages.

### 4.6. Cell Culture

To test the ability of the extracts to inhibit LPS-stimulated nitric oxide production, the murine macrophage cell line RAW 264.7 was used. Cells were grown in plastic culture flasks in DMEM supplemented with 1% L-glutamine, 10% FBS, and 1% antibiotic/antimycotic solution (penicillin/streptomycin) under 5% CO_2_ conditions at 37 °C. After 4–5 days, the cells were removed from the culture flask by scraping and centrifugation for 10 min at 1500 rpm. The medium was then removed, and the cells were resuspended with fresh DMEM. Cell counts and viability were determined using a standard trypan blue cell counting technique. Cell monolayers were subcultured into 96-well culture plates (1 × 10^5^ cells/well) and used for experiments after 24 h.

#### In Vitro Evaluation of the Inhibitory Properties on Nitric Oxide (NO) Production 

The presence of nitrite, a stable oxidized product of NO, was determined in cell culture media by Griess reagent (1% sulfonamide and 0.1% *N*-(1-naphtyl) ethylenediamine dihydrochloride in 2.5% H_3_PO_4_). Briefly, 100 μL of cell culture supernatant was removed and combined with 100 μL of Griess reagent in a 96-well plate followed by spectrophotometric measurement at 550 nm using a microplate reader (GDV DV 990 B/V, Rome, Italy). Cytotoxicity was determined using the MTT assay. The absence of cytotoxic effects on RAW 264.7 macrophages was verified by the MTT assay. At the end of the experiments, MTT (0.5%, 100 μL/well) was added to the wells. Four hours later, MTT was removed, and 100 μL/well of DMSO was added, and the absorbance was measured at 550 nm.

### 4.7. Measurement of Pancreatic Lipase and α-Amylase Activities

The inhibition of pancreatic lipase was determined as described by Grande F. and colleagues [27]. An aqueous solution (1 mg mL^−1^) of crude porcine pancreatic lipase type II and a 5 mmol L−1 solution of 4-nitrophenyl octanoate (NPC) in dimethyl sulfoxide was prepared. In a test tube, 100 µL of 5 mmol L^−1^ of NPC, 4 mL of Tris-HCl buffer (pH = 8), 100 µL of extract/fraction, and 100 µL of enzyme solution were mixed. The mixture was incubated at 37 °C for 25 min before the substrate was added. In the control, the extract was replaced with the same volume of dimethyl sulfoxide. The absorbance was measured in cuvettes at 412 nm. A blank sample without the enzyme was measured for each extract. For comparison, orlistat was tested at a final concentration of 20 µg mL^−1^. Pancreatic α-amylase inhibition was performed by the DNSA chromogenic method, based on the measurement of maltose generation, quantified by the reduction of 3,5-dinitrosalicylic acid to 3-amino-5-nitrosalicylic acid. A starch solution (0.5% *w*/*v*) was obtained by stirring potato starch in sodium phosphate buffer 0.02 mM with 6.7 mM sodium chloride (pH = 7). A solution of α-amylase 0.5 mg/mL was prepared in cold distilled water. A dinitrosalicylic acid (DNSA) solution was prepared by dissolving 15 g of Rochelle salt, 15 mL of 2 N NaOH, and 0.5 g of 3,5-dinitrosalicylic acid in 50 mL of distilled water. In a test tube, 250 µL of extract/fraction was mixed with 250 µL of α-amylase solution and incubated at 37 °C for 10 min. Then, 250 µL of starch solution was added, and the mixture was incubated for another 15 min. Then, 2 mL of DNSA reagent was added, and the mixture was incubated at 85 °C for 15 min. The reaction was then cooled and diluted with 15 mL of distilled water. The absorbance was measured at 540 nm. A blank sample without the enzyme was measured for each extract. Acarbose was used as a positive control. A control without extract/acarbose was prepared. All the tests were performed in triplicate.

## 5. Conclusions

In conclusion, the results obtained in the present study demonstrate that extracts of the branches and apical shoots of *Pinus nigra* subsp. *laricio* (Poir.) Maire possess significant antioxidant and energy metabolism modulator potential. The traditional use of larch pine in folk medicine, in combination with our results, suggests that this tree species may represent a rich source of bioactive compounds with potential health benefits.

Although further investigation is needed to identify the bioactive compounds responsible for the activities and elucidate the molecular mechanisms underlying these effects, our data support the hypothesis that larch pine extracts may represent a new class of therapeutic agents for modulating energy metabolism, with possible implications in body weight management.

## Figures and Tables

**Table 1:** Extraction yields and total phenolic and flavonoid contents.

Sample	Yield (%)	TPC ^1^	TFC ^2^
Apical shoots	10.88	20.66 ± 0.27 ^a^	0.40 ± 0.01 ^a^
Branches	6.40	18.10 ± 0.70 ^b^	0.24 ± 0.01 ^b^

^1^ Total phenolic content. ^2^ Total flavonoid content. Data are expressed as mean ± SEM (*n* = 3). Results were expressed as mg of chlorogenic acid and quercetin equivalent per g of dry plant material for the phenolic and flavonoid contents, respectively. Different letters along columns (TPC and TFC) indicate statistically significant differences at *p* < 0.05 (Student’s *t*-test)

**Table 2:** Apolar compounds identified in the *n*-hexane fractions.

Compound ^1^	Rt ^2^	RAP ^3^	
		Apical Shoots	Branches
Borneol	9.98	Tr ^4^	-
2-Decenal	10.95	Tr	-
Bornyl acetate	11.21	Tr	-
Cyclotetradecane	12.12	Tr	-
Phenethyl isovalerate	13.03	Tr	-
Hexadecene	13.73	Tr	-
Pristanic acid	15.52	0.1	-
Pentadecanoic acid, 14 methyl-, methyl ester	16.02	0.7	-
Palmitic acid	16.07	1.1	1.0
Manoyl oxide	16.69	0.5	-
Margaric acid	16.93	0.3	0.3
10,13-Octadecadienoic acid, methyl ester	17.07	0.3	-
Vaccenic acid	17.14	-	3.7
Ethyl linoleate	17.52	0.6	2.5
Elaidic acid	17.55	-	6.2
Linoleic acid	17.91	0.4	2.0
3-(4-N, N-dimethylaminophenyl) propenoic acid 2-(diethoxyphosphinyl)-ethyl ester	19.60	1.8	2.9
Cyclotetracosane	20.61	-	1.0
9-Octadecenal	20.67	-	5.2
γ-Sitosterol	22.94	0.26	2.0

^1^ Compounds listed in order of elution from SE30 MS column. ^2^ Retention time (in min). ^3^ Relative area percentage (peak area relative to total peak area in total ion current (TIC) %). ^4^ Tr: trace percentage < 0.1%.

**Table 3:** Compounds identified in dichloromethane fraction.

Compound ^1^	Rt ^2^	RAP ^3^	
		Apical Shoots	Branches
Benzoic acid	10.25	-	0.6
1-Tetradecene	12.13	0.18	-
Vanillyl alcohol	12.84	-	0.1
1-Hexadecene	13.74	0.37	-
1-Octadecene	15.15	0.24	-
Cetene	15.79	-	0.2
Hexadecanoic acid, methyl ester	16.02	1.17	-
Hexadecanoic acid, ethyl ester	16.33	0.99	-
5-Octadecene	17.00	1.91	-
Isomaturnin	17.06	-	0.4
Decanedioic acid, dibutyl ester	17.50	1.33	-
2(1H)-Naphthalenone,octahydro-4a-phenyl	17.78	-	0.4
Pinocembrin	19.60	-	0.5
Stigmast-3-en-6-ol	22.66	3.2	-

^1^ Compounds listed in order of elution from SE30 MS column. ^2^ Retention time (in min). ^3^ Relative area percentage (peak area relative to total peak area in total ion current (TIC) %).

**Table 4:** IC_50_ values of antioxidant activity.

Sample	Fraction	IC_50_ (µg/mL)		
		DPPH Test	β-Carotene *Bleaching* Test	
			30 min	60 min
Apical shoots	Raw extract	326.20 ± 1.10 ^f^	35.27 ± 1.32 ^d,e^	46.49 ± 1.10 ^f^
	*n*-Hexane	>1000	>100	>100
	CH_2_Cl_2_	>1000	92.32 ± 1.58 ^i^	>100
	AcOEt	196.10 ± 3.50 ^d^	10.69 ± 0.26 ^b^	23.51 ± 0.85 ^c^
	H_2_O	>1000	>100	>100
Branches	Raw extract	26.14 ± 0.90 ^b^	3.33 ± 0.16 ^a^	5.07 ± 0.15 ^a^
	*n*-Hexane	666.73 ± 9.90 ^g^	60.25 ± 0.63 ^g^	88.20 ± 1.96 ^i^
	CH_2_Cl_2_	271.27 ± 8.23 ^e^	38.27 ± 0.78 ^e^	68.66 ± 1.83 ^h^
	AcOEt	15.67 ± 0.16 ^a,b^	11.35 ± 0.15 ^b^	33.54 ± 1.26 ^d^
	H_2_O	60.72 ± 1.78 ^c^	36.39 ± 0.84 ^d,e^	92.26 ± 3.35 ^i^
Ascorbic acid *		2.00 ± 0.01 ^a^	-	-
Propyl gallate *		-	1.00 ± 0.02 ^a^	1.00 ± 0.02 ^a^

Data are expressed as mean ± S.D.M (*n* = 3). Different letters along columns (DPPH) or between columns (β-carotene bleaching test) indicate statistically significant differences at *p* < 0.05 (Bonferroni post hoc test). * Positive controls.

**Table 5:** Nitric oxide inhibition induced on macrophage cells.

Sample	Fraction	IC_50_ (μg/mL)
Apical shoots	Raw extract	n.a.
	*n-*Hexane	84.40 ± 1.54 ^b^
	CH_2_Cl_2_	n.a.
	AcOEt	n.a.
	H_2_O	n.a.
Branches	Raw extract	213.20 ± 6.14 ^c^
	*n*-Hexane	43.52 ± 2.34 ^a^
	CH_2_Cl_2_	50.68 ± 3.63 ^a^
	AcOEt	n.a.
	H_2_O	n.a.
Indomethacin *		53.00 ± 0.81 ^a^
L-NAME *		45.86 ± 0.46 ^a^

Data are expressed as mean ± S.D.M. (*n* = 4). Different letters indicate statistically significant differences at *p* < 0.05 (Bonferroni post hoc test). * Positive controls.

**Table 6:** IC_50_ values of inhibition of pancreatic lipase of the extracts under examination.

Sample	Fraction	IC_50_ (mg/mL)
Apical shoots	Raw extract	1.33 ± 0.05 ^c^
	H_2_O	4.20 ± 0.18 ^d^
Branches	Raw extract	0.62 ± 0.02 ^b^
	H_2_O	n.a.
Orlistat *		0.018 ± 0.001 ^a^

Data are expressed as mean ± S.D.M. (*n* = 3). Different letters indicate statistically significant differences at *p* < 0.05 (Bonferroni post hoc test). * Positive controls.

**Table 7:** IC_50_ amylase inhibition values obtained.

Sample	Fraction	IC_50_ (µg/mL)
Apical shoots	Raw extract	139.00 ± 1.50 ^d^
	*n*-Hexane	198.17 ± 2.15 ^f^
	CH_2_Cl_2_	161.90 ± 1.61 ^e^
	AcOEt	22.05 ± 0.29 ^b^
	H_2_O	47.32 ± 1.43 ^c^
Acarbose *	-	12.68 ± 0.54 ^a^

Data are expressed as mean ± S.D.M. (*n* = 3). Different letters indicate statistically significant differences at *p* < 0.05 (Bonferroni post hoc test). * Positive controls.

## Data Availability

Data are contained within the article.
